# Retraction Note: Inhibition of inflammation using diacerein markedly improved renal function in endotoxemic acute kidney injured mice

**DOI:** 10.1186/s11658-023-00524-y

**Published:** 2024-01-08

**Authors:** Guangzhe Yu, Qian Liu, Xuening Dong, Kaihong Tang, Bohui Li, Chunmei Liu, Wenzheng Zhang, Yiduo Wang, Yingyu Jin

**Affiliations:** 1 Department of Emergency Surgery, The 1st Affiliated Hospital of Harbin Medical University, Harbin, Heilongjiang Province China; 2 Department of Laboratory Diagnosis, The 1st Affiliated Hospital of Harbin Medical University, 23 Youzheng Street, Nangang District, Harbin, 150001 Heilongjiang Province People’s Republic of China

**Retraction: Cellular & Molecular Biology Letters (2018) 23:38** 10.1186/s11658-018-0107-z

The Editor-in-Chief has retracted this article due to concerns about several images, specifically:

- In Fig. 1C the LPS panel is similar to Fig. 6C WKY/BCL6 OE in a previously-published paper by different authors [1].
- In Figs. 2A,B there are multiple overlaps with panels in Figs. 8 and 3A in two previously-published papers by different authors [2, 3] respectively.

The authors stated that they used third-party services to obtain some of their data. The Editor-in-Chief, therefore, has lost confidence in the integrity of the article's findings. We contacted the authors on the emails they provided at submission. Yingyu Jin has stated on behalf of the authors that they agree to this retraction.
